# Five-Year Clinical and MRI-Based Outcomes After Cartilage Repair With or Without ACL Reconstruction: Worse Clinical Outcome after ACL Reconstruction Does not Affect Cartilage Repair Tissue Quality

**DOI:** 10.1177/19476035251362433

**Published:** 2025-08-08

**Authors:** Felix R.M. Koenig, Veronika Vetchy, Gregor Wollner, Maximilian Kern, Marcus Raudner, Veronika Janacova, Vladimir Juras, Pavol Szomolanyi, Markus M. Schreiner, Siegfried Trattnig

**Affiliations:** 1 High-Field MR Centre, Department of Biomedical Imaging and Image-Guided Therapy, Medical University of Vienna, Vienna, Austria; 2 Department of Biomedical Imaging and Image-Guided Therapy, Medical University of Vienna, Vienna, Austria; 3 Department of Orthopedics and Trauma-Surgery, Medical University of Vienna, Vienna, Austria; 4 Department of Imaging Methods, Institute of Measurement Science, Slovak Academy of Sciences, Bratislava, Slovakia; 5 Austrian Cluster for Tissue Regeneration, Vienna, Austria; 6 Institute for Clinical Molecular MRI in the Musculoskeletal System, Karl Landsteiner Society, Vienna, Austria

**Keywords:** clinical research cartilage and bone tissue engineering, magnetic resonance imaging, clinical research knee, basic cartilage and bone imaging, ligament studies (instability)

## Abstract

**Objectives:**

Cartilage repair (CR) surgery and anterior cruciate ligament reconstruction (ACL-R) are common joint procedures, particularly in younger patients. However, the impact of prior or concurrent ACL-R on the outcomes of CR remains uncertain. This study aimed to evaluate whether ACL-R affects the structural quality and clinical outcomes of CR tissue.

**Methods:**

In this retrospective multicenter study, 71 patients undergoing CR were followed up with magnetic resonance imaging (MRI) and clinical evaluations at 3, 12, and 60 months. Of these, 22 patients underwent ACL-R before or during CR. Morphological assessment was performed using Magnetic Resonance Observation of Cartilage Repair Tissue (MOCART) 2.0 scores; compositional analysis included T2 mapping (*n* = 45). Patient-reported outcome measures (PROM) were assessed at all time points. Statistical tests included the Mann–Whitney *U*-test, Wilcoxon signed-rank test, and simulation-based power analysis.

**Results:**

MOCART 2.0 scores and T2 mapping values showed no significant group differences at any time point. Both groups demonstrated significant improvements in PROMs from baseline to 60 months. However, at 60 months, the ACL-R group had significantly lower PROMs than the non-ACL-R group.

**Conclusion:**

Although long-term clinical outcomes were worse in patients with ACL-R, all PROMs improved significantly from baseline to 60 months in both groups. MRI showed no significant differences in focal CR tissue quality, suggesting structural success regardless of ACL-R. While ACL-R patients remain at higher risk of joint degeneration, they can still experience mid-term clinical benefit from CR. These findings support its use in ACL-R patients when joint function is properly restored and expectations are managed.

## Key Points

- MRI-based morphological and compositional assessments revealed no significant differences in CR tissue between the groups.
- Patients with ACL-R demonstrated significantly worse long-term clinical outcomes after CR compared to those without.
- These findings support the use of CR in ACL-R patients while emphasizing the need for biomechanical stability to mitigate the risk of long-term joint degeneration.

## Introduction

The articular cartilage, subchondral lamina, and subchondral bone form a crucial functional unit that is receiving increasing attention for their important role in preserving joint health.^1^ Cartilage repair (CR) surgery aims to restore this unit, and procedures such as Microfracturing (MFX) and Matrix-Induced Autologous Chondrocyte Implantation (MACI) are frequently used, particularly in younger patients.^2^ However, it remains unclear which potential co-factors may negatively influence post-operative clinical outcomes.

The anterior cruciate ligament (ACL) serves a crucial function in stabilizing the knee by limiting the forward movement of the tibia and controlling rotational forces within the tibiofemoral joint.^3^ Ruptures of the ACL and consecutive anterior cruciate ligament reconstruction (ACL-R) are frequently performed surgery, in particular in young patients.^4^ This procedure is primarily undertaken to restore stability following ACL injury and may reduce the risk of secondary meniscal or cartilage damage, which are considered contributing factors in the development of osteoarthritis (OA).^5,6^ However, the literature is conflicting, as other clinical trials have shown that ALC-R does not prevent post-traumatic OA compared to rehabilitation.^7,8^

While CR surgery and ACL-R are widely performed, their combined long-term effects remain unclear. The key clinical question addressed in this study is whether patients with prior ACL reconstruction benefit from focal CR, and whether ACL-R influences the structural or compositional integrity of the repaired cartilage tissue. While it is well established that ACL-R patients are at increased risk of developing OA in the long term, it remains unclear whether focal CR can provide meaningful functional improvements in this population, and whether altered biomechanics affect CR success.

Therefore, the aim of this study was to compare clinical, morphological, and compositional MRI outcomes—including T2 mapping—in patients with and without ACL-R over a 5-year follow-up. The goal was to evaluate whether CR can offer mid- to long-term clinical benefit in ACL-R patients and to assess potential interactions between ACL-R and focal CR. This study is intended to support orthopedic decision-making by clarifying whether CR is structurally successful in the ACL-R population and to help inform patients about their mid- to long-term prognosis.

## Methods

### Study Design and Inclusion Criteria

This study retrospectively analyzed the clinical, MR morphological, and compositional outcomes of patients who had previously undergone ACL-R after CR in comparison to patients without ACL-R.

Patients were enrolled in a multicenter, randomized, controlled, open-label (with blinded MRI reading) study and provided signed informed consent after being fully informed. The study was conducted in full compliance with the principles laid down in the Declaration of Helsinki, the ICH E6 Guideline for Good Clinical Practice, and all applicable local laws and regulations. Approval was obtained from the local ethics committees and federal authorities at each participating site prior to patient enrollment. The study was conducted from May 2013 to June 2023 to compare the outcomes after MFX and MACI. This trial was registered under EudraCT number 2011-005798-22 and ClinicalTrials.gov identifier NCT01656902.

The inclusion criteria were as follows: a cartilage defect grade III or IV according to the International Cartilage Repair Society (ICRS) with a size of 2 to 6 cm^2^ in either the femoral condyle or the trochlea of the knee was the base criterion for inclusion in the study. All included patients had a clinically stable knee joint or adequately reconstructed ligaments at the time of CR, as determined by the treating surgeon, in accordance with the study’s inclusion criteria. If not, ligament repair must be performed before, during, or within 6 weeks of cartilage treatment. So patients in the ACL-R group underwent ACL-R either prior to, simultaneously with, or within 6 weeks of CR. The patient has a full range of motion in the affected knee joint or a loss of no more than 10° in extension and flexion. The patient’s meniscus is undamaged, with up to 50% resection is permitted.

The exclusion criteria included the following: inability to undergo specific medical imaging, previous CR surgeries, degenerative joint diseases with a Kellgren and Lawrence grade of 2 or higher, inflammatory arthritis, malalignment (specifically valgus- or varus deformity), systemic diseases, infections, a body mass index (BMI) over 35 kg/m^2^, substance abuse, cognitive impairments, active systemic or local microbial infection at the surgical site, and a known history of cancer within the past 5 years. Patients with a documented ACL-graft failure or revision ACL reconstruction were excluded from the study.

Written and informed consent was obtained from all patients included in this study.

### Magnetic Resonance Examination

All examinations for this investigation were conducted using a 3 Tesla whole-body magnetic resonance (MR) scanner. MRI scans were conducted using Siemens 3T MRIs from Siemens Healthineers in Erlangen, Germany, Philips Medical Systems 3T MRIs from Philips Medical Systems in Best, the Netherlands, and a GE Medical Systems 3T MRI from GE Medical Systems in Chicago, Illinois. This multicenter study utilized a range of 8 to 16 knee channel coils. The detailed presentation of the comprehensive MR examination methodology and its corresponding sequence parameters can be seen in **Table 1**.

**Table 1.** Specifications of the MRI Protocol Employed in This Multicenter Trial.

Parameter	T2 Mapping	TSE PD	TSE T2w	SE T1w
*Orientation Plane*	*Sagittal*	*Coronal*	*Sagittal*	*Sagittal*
Slice thickness (mm)	3	3	2	2
Slice spacing (mm)	3.3	3.3	2.2	2.2
Repetition time (ms)	2000	3080	3310	700
Echo time (ms)	12.5; 25; 37.5; 50; 62.5; 75; 87.5; 100	28	12	12
Averages	1	2	3	1
Flip angle (°)	90, 180	180	180	90
Acquisition matrix	320 × 256	448 × 403	381 × 448	448 × 381
Image matrix	320 × 320	896 × 896	448 × 448	448 × 448
Field of view (cm)	16 × 16	16 × 16	16 × 16	16 × 16
Total acquisition time (min:s)	10:36	04:46	03:55	03:53

The study procedure consisted of morphological components, which involved the use of Turbo Spin Echo (TSE) Proton Density (PD), TSE T2-weighted (w), and SE T1w sequences. The accuracy and reproducibility of these sequences for cartilage morphology evaluation have been validated in prior multicenter studies and are routinely used in clinical cartilage imaging.^9^ In addition, there was a compositional component that utilized T2 mapping with a multi-echo multi-slice sequence. The T2 maps were acquired using the parameters specified in **Table 1** and have already been described in other studies of our research group.^10^

All images were sent to the primary reading center, where T2 maps were generated using a 2-parametric exponential fitting method, and CR segmentation and scoring were performed.

### Image Analysis (T2 Mapping and MOCART Score 2.0)

**Figure 1.**
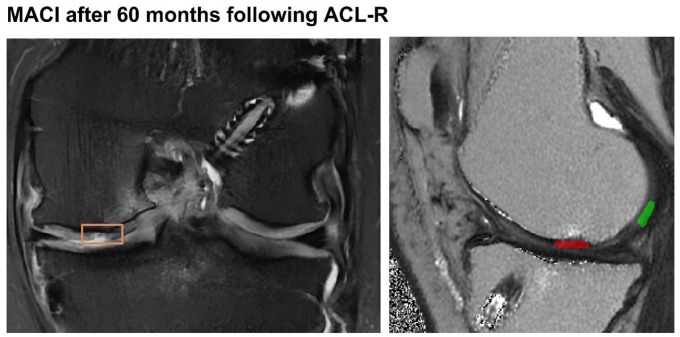
60 months after cartilage repair following ACL-R. The left image shows an MR tomographic image, showing incomplete filling of the cartilage transplant on a PD fs sequence. The right image shows the associated T2 map in a sagittal view. T2 ratio was calculated between the T2 values of the treated area (green) and the reference cartilage of the femur (green). Both images were obtained using the image analysis tool ITK-SNAP.

The T2 values were evaluated using ITK-Snap^11^ by the authors M.R. (senior radiologist with over 8 years of experience in MSK imaging) and F.R.M.K. (resident radiologist with 3 years of experience in MSK Imaging). At least 3 regions of interest (ROIs) were defined within CR. The CR sites were assessed on 3 consecutive sagittal sections in each case. To obtain specific reference values for the T2 measurements of each patient, we identified femoral cartilage that had no significant morphological abnormalities, had the same orientation as the graft tissue (to eliminate magic angle effect), and was not in close proximity to the repaired tissue. We then delineated 3 distinct ROIs in at least 3 consecutive sagittal slices, as shown in **Figure 1**. The reproducibility of T2 mapping has been demonstrated in numerous prior investigations.^12,13^

The average T2 values of the restored tissue were divided by the average T2 values of the morphologically inconspicuous cartilage in the femur. The global T2 ratio, generated in this manner, was used for statistical analysis.

The MOCART 2.0 (Magnetic Resonance Observation of Cartilage Repair Tissue 2.0) was used to assess the morphological result of a CR process in a semi-quantitative manner.^14^ The purpose of this technique was to methodically record the condition of the CR site and the tissues around it. The reliability, repeatability, and usefulness of this methodology in various surgical cartilage restoration methods have been extensively proven.^15,16^

The scoring system consists of 7 variables, each contributing to a maximum score of 100. These subscores include the volume fill of the cartilage defect, integration into the adjacent cartilage, surface characteristics of the repair tissue, structure of the repair tissue, signal intensity of the repair tissue, presence of bony defect or bony overgrowth, and subchondral changes. The assessments were carried out by a highly experienced senior musculoskeletal radiologist with over 30 years of expertise in the domain. The MR tests were conducted after 3, 12, and 60 months after the CR procedure.

Fat-suppressed PD MRI sequences in the coronal plane were retrospectively used to assess whether a patient received ACL reconstruction.

Patients after MACI and MFX were included. MFX is a minimally invasive surgical technique that involves creating small fractures in the subchondral bone to stimulate the formation of fibrocartilage for cartilage defect repair. MACI involves harvesting and expanding the patient’s chondrocytes, which are then seeded onto a scaffold and implanted into the defect. MACI promotes the regeneration of hyaline-like cartilage, offering a durable and functional repair. Both techniques provide valuable options for CR, with MACI showing promise for larger and more complex lesions.^17^

### Clinical Examination

Clinical outcomes were assessed using patient-reported outcome measures (PROMs), specifically the International Knee Documentation Committee (IKDC) and Knee Injury and Osteoarthritis Outcome Score (KOOS). In accordance with the multi-center trial protocol, all observers at the participating sites performed evaluations in a blinded manner, following standardized procedures to ensure independent and unbiased assessment.

The KOOS is a self-administered survey that evaluates 5 specific areas of knee function: pain, symptoms, everyday activities, sports and recreational activities, and overall quality of life. The IKDC is a survey conducted by a doctor to evaluate the overall functionality of the knee. These scores were utilized in accordance with established protocols. This study includes scores assessed at baseline prior to the intervention, as well as at 3, 12, and 60 months after the intervention.

### Statistical Evaluation

The statistical analysis was conducted by a biomedical statistician using IBM SPSS version 24.0.1 for Windows (IBM, Chicago, Illinois). Data metrics are usually represented by the mean value along with the standard deviation (SD). The images were generated using GraphPad Prism version 10 (GraphPad Software, La Jolla, California; www.graphpad.com) and SPSS.

As this was a retrospective study, no *a priori* sample size calculation was performed. A simulation-based power analysis (10,000 iterations) was performed in R (Version 4.2.1; R Core Team, 2022), using the observed differences and SD at 60 months for KOOS, IKDC, and MOCART 2.0 scores. Power calculations were based on actual group means and pooled variances.

Before conducting Mann–Whitney *U*-test and Wilcoxon rank test, we assessed the normality of the data using the Shapiro–Wilk test, a non-parametric test for normality. The results indicated that the data were not normally distributed (*P* < 0.05). A significance level of 0.05 or less was used to indicate statistical significance.

## Results

### Patient Characteristics

**Figure 2.**
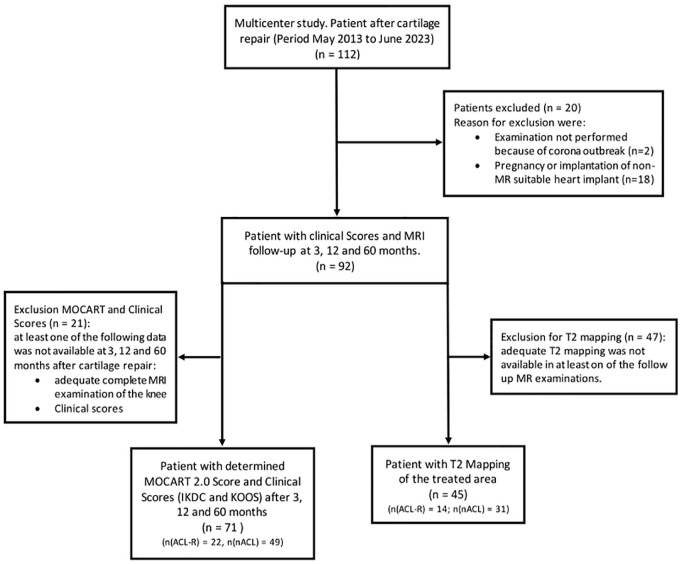
Study flowchart of this retrospective analysis. ACL-R = anterior cruciate ligament-reconstruction; nACL = no anterior cruciate ligament reconstruction.

A flowchart of this retrospective study is shown in **Figure 2**. At the time of this study, clinical and MR follow-up data were available for 112 patients at our research site. Adequate and complete MR examinations and clinical scores were available for 71 patients. ACL-R was performed in 22 patients (31.0%) prior, during, or within 6 weeks after CR (MACI *n* = 16, MFX *n* = 6). The control group consisted of 49 patients (69.0%) (MACI *n* = 30, MFX *n* = 19). T2 maps were available for 45 patients who were part of the MRI part of the study. These patients had T2 maps and underwent MRI at all follow-up visits (3, 12, and 60 months after CR surgery). Of the total, 14 individuals (31.1%) had ACL-R prior to CR (MACI *n* = 11, MFX *n* = 3) and 31 individuals (68.9%) had no ACL-R prior to and during the follow-up interval (MACI *n* = 17, MFX *n* = 14).

Retrospectively, 71 participants were included, of whom 21 (29.6%) were identified as female and 50 (70.4%) as male. The mean age at intervention was 38 years (SD = 10.6), ranging from 17 to 56 years. Demographic comparisons revealed that the ACL-R group was significantly younger than the no-ACL-R group (34.05 ± 10.19 vs. 39.78 ± 10.43 years, *P* = 0.035) and included a higher proportion of male patients (90.9% vs. 61.2%). Due to incomplete documentation, BMI data were not evaluated. Post-debridement defect size was comparable between the ACL-R group (3.08 ± 0.77 cm^2^) and the no-ACL-R group (3.22 ± 0.92 cm^2^, *P* = 0.794). Pre-debridement defect size was also similar between groups, with a mean of 2.98 ± 0.82 cm^2^ in the ACL-R group and 3.01 ± 1.03 cm^2^ in the no-ACL-R group (*P* = 0.843). While a clinical history of meniscal injury was unavailable, no meniscal tears were detected on follow-up MR imaging in either group.

### MOCART Score 2.0

**Figure 3.**
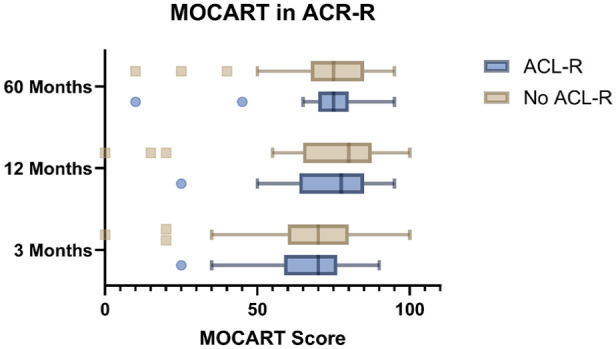
Comparison of MOCART scores in ACL-R and non-ACL-R groups over time: no significant differences were seen between the groups.

Despite slightly higher mean ranks for the no-ACL-R group at each time point (MOCART 3 months: 36.32 vs. 35.30; MOCART 12 months: 37.31 vs. 33.09; MOCART 60 months: 36.07 vs. 35.84), statistical tests revealed no significant difference between the groups, with *P*-values of 0.846, 0.423, and 0.965, respectively (**Figure 3** and **Table 2**).

**Table 2.** MOCART Score After 3, 12, and 60 Months After Cartilage Repair Categorized in ACL-R and no ACL-R.

	MOCART_Total_3M	MOCART_Total_12M	MOCART_Total_60M
Mean ± SD	No-ACL-R: 67.35 ± 19.178	No-ACL-R: 75.10 ± 20.6	No-ACL-R: 74.39 ± 17.1
	ACL-R: 67.50 ± 15.868	ACL-R: 73.41 ± 16.7	ACL-R: 73.86 ± 17.6
Median + Interquartile Range (IQR)	No-ACL-R: 70.00 (IQR = 20)	No-ACL-R: 80.00 (IQR = 23)	No-ACL-R: 75.00 (IQR = 18)
	ACL-R: 70.00 (IQR = 18)	ACL-R: 77.50 (IQR = 21)	ACL-R: 75.00 (IQR = 10)

### Clinical Scores

KOOS and IKDC scores were assessed before surgery and at 3, 12, and 60 months after surgery. All scores improved significantly compared to baseline in both groups:

In the “ACL-R” group, the IKDC score improved from 39.3 at baseline to 64.0 at 60 months (*P* < 0.001), and the KOOS score increased from 47.1 at baseline to 69.7 at 60 months.

Significant improvements in all KOOS dimensions were shown in all subscores with *P* < 0.001 (pain, symptoms, daily function, sport function, and quality of life).

In the “No ACL-R” group, the IKDC score improved from 37.9 at baseline to 79.5 at 60 months (*P* < 0.001), and the KOOS score increased from 49.8 to 85.5. There was a significant improvement in all dimensions of the KOOS score, with *P* < 0.001 in all subscores (pain, symptoms, daily function, sports function, and quality of life).

Absolute scores of both PROMs at 60 months were significantly higher in the no-ACL-R group (“No ACL-R” vs. “ACL-R”: IKDC: 79.5 vs. 64.0; KOOS: 85.5 vs. 69.7).

**Figure 4.**
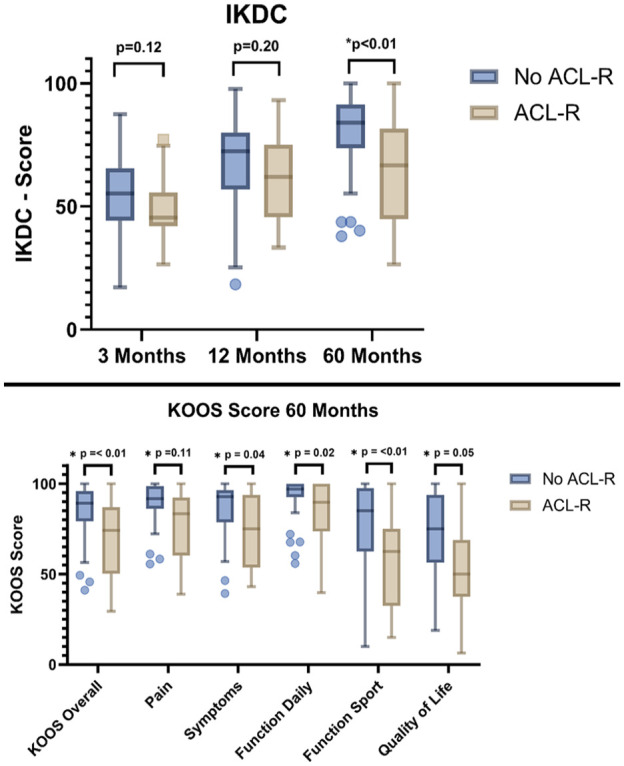
Significant differences in IKDC score after ACL-R (mean 64 points) and no ACL-R group (mean 79 points). All subscores of the KOOS score were also significantly lower in the ACL-R group.

Differences between patients with and without ACL reconstruction are shown in **Table 3** and **Figure 4**. Significant differences were found in all 5 KOOS dimensions and in the IKDC score after 60 months. When compared to the published PASS thresholds (IKDC ≥75.9; KOOS subscales: pain ≥88.9, symptoms ≥57.1, daily function ≥91.7, sports ≥75.0, quality of life ≥62.5), only the no-ACL-R group exceeded these benchmarks across all scores, while the ACL-R group did not reach any of the thresholds.^18^ No significant differences were seen in PROMs at baseline, 3 months, or 12 months between the groups.

**Table 3.** Clinical Scores (IKDC and KOOS) at Baseline and 3, 12, and 60 Months After Cartilage Repair, Categorized With and Without ACL-R.

	No ACL-R	ACL-R	*P*
KOOS baseline	49.80 ± 15.76	47.13 ± 15.79	0.78
Pain baseline	64.51 ± 17.66	57.62 ± 15.85	0.28
Symptoms baseline	60.88 ± 17.86	58.86 ± 18.62	0.79
Function daily baseline	67.15 ± 19.46	63.10 ± 19.04	0.66
Function sport baseline	26.36 ± 23.26	28.54 ± 20.03	0.41
Quality life baseline	30.13 ± 15.65	30.08 ± 18.51	0.99
KOOS 3 months	61.41 ± 15.18	56.79 ± 15.87	0.17
Pain 3M	75.52 ± 13.68	71.80 ± 15.66	0.27
Symptoms 3M	68.46 ± 16.48	67.86 ± 16.75	0.79
Function daily 3M	80.38 ± 14.60	75.28 ± 15.81	0.14
Function sport 3M	37.26 ± 23.22	31.82 ± 23.17	0.32
Quality life 3M	45.45 ± 19.64	40.63 ± 18.80	0.37
KOOS 12 months	75.92 ± 14.28	69.69 ± 17.69	0.19
Pain 12M	83.83 ± 12.34	80.30 ± 14.86	0.55
Symptoms 12M	78.80 ± 15.19	72.73 ± 19.39	0.23
Function daily 12M	88.94 ± 1.76	86.29 ± 3.18	1.00
Function sport 12M	60.23 ± 26.53	63.13 ± 22.38	0.78
Quality Life 12M	53.43 ± 24.0	60.69 ± 23.74	0.27
KOOS 60 months	84.99 ± 14.97	69.66 ± 20.25	**<0.01** *
Pain 60M	90.03 ± 1.57	79.04 ± 3.86	**0.01** *
Symptoms 60M	86.42 ± 2.10	75.17 ± 4.25	**0.04** *
Function daily 60M	93.59 ± 1.56	84.02 ± 3.68	**0.02** *
Function sport 60M	77.39 ± 3.47	54.55 ± 5.47	**<0.01** *
Quality life 60M	73.33 ± 3.14	53.43 ± 5.60	**0.05** *
IKDC baseline	37.87 ± 1.90	39.29 ± 3.09	0.80
IKDC 3 months	54.06 ± 2.20	49.68 ± 2.91	0.12
IKDC 12 months	68.54 ± 2.57	62.96 ± 3.86	0.20
IKDC 60 months	79.47 ± 2.39	63.98 ± 4.64	**0.01** *

Significantly lower scores were seen in the ACL-R group after 60 months in both scores. Significant values are marked with *.

### T2 Mapping

**Figure 5.**
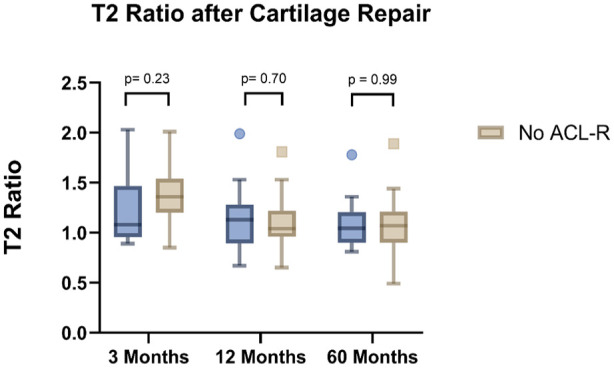
No significant differences were seen in the T2 ratio between ACL-R and no ACL-R after cartilage repair.

Adequate T2 mapping was available in 45 patients (ACL-R *n* = 14, no-ACL-R *n* = 31). There were no significant differences in the T2 ratio of the treated area at 3, 12, and 60 months among patients with or without ACL-R at the respective time intervals (*P*-value after 3 months = 0.23, 12 months = 0.70, 60 months = 0.99). Findings are summarized in **Table 4** and shown in **Figure 5**.

**Table 4.** T2 Ratio of the Treated Cartilage Area.

Time	Mean T2 Ratio ACL-R	Mean T2 Ratio No ACL-R	*P*
3 months	1.21 (SD: 0.36)	1.36 (SD: 0.30)	0.23
12 months	1.09 (SD: 0.23)	1.15 (SD: 0.33)	0.7
60 months	1.06 (SD: 0.26)	1.09 (SD: 0.27)	0.99

There were no significant differences between the groups.

### Simulation-Based Power Analysis

Regarding the statistical power, a simulation-based power analysis using the observed mean difference of 15 points for KOOS scores (pooled SD = 17) and 15.5 points for IKDC scores (pooled SD = 19.1) demonstrated a statistical power of 0.92 (KOOS) and 0.87 (IKDC). For the MOCART 2.0 score, a mean difference of 15 points (which is considered a criterion for a clinically significant difference, as shown in previous studies^19^) was applied. With the highest observed SD of 17.1, the analysis demonstrated a statistical power of 0.98.

## Discussion

ACL-R and CR are both commonly performed in young, active patients. The main finding of our study is that, over a 5-year follow-up, no significant differences were observed in the focal morphological or compositional MRI characteristics of the CR tissue between patients with and without prior ACL-R. This confirms our hypothesis that ACL-R does not impair the structural integration of repaired cartilage.

However, patients with prior ACL-R had significantly worse clinical outcomes, indicating that while CR tissue quality remains intact, ACL-R negatively influence overall joint function, likely due to global biomechanical factors. Thus, the structural aspect of our hypothesis was confirmed, while the clinical aspect revealed important differences. To our knowledge, this is the first study to evaluate the influence of ACL-R on CR in a comparable patient cohort using long-term morphological and compositional MRI analysis. For the evaluation of the morphological outcome after CR, the MOCART score 2.0 is one of the most commonly used MRI scoring systems for morphological assessment of CR tissue.^14,15^ While other studies have focused on global knee outcomes, this study uniquely shows no significant focal MRI measureable effects of ACL-R on the CR tissue, challenging some previous hypotheses about biomechanical aberration.

Previous research by Su *et al*. has demonstrated that T2 values in the central aspect of the medial femoral condyle after ACL-R are significantly elevated in comparison to non-treated controls as a sign of early degenerative cartilage disease. The group demonstrated T2 mapping as a powerful tool to evaluate early changes in the cartilage matrix.^20^ In this study, we focused on the possible focal effect of ACL-R on CR tissue and could not demonstrate any focal MR morphological and compositional effects on CR tissue. In a 2010 review article, Georgoulis *et al*. addressed the issue of aberrant biomechanical patterns after ACL-R, which may lead to stress on focal cartilage regions that are typically not affected by such loads in a healthy knee.^21^ They recommended further experimental work, to develop new surgical procedures that imitate the natural ACL anatomy more closely.^21^ However, these abnormal biomechanical patterns seem not to influence the CR area significantly 3 months postoperatively and in the long-term after 60 months as shown in our study.

We showed significant differences in clinical scores after CR surgery in favor of patients without ACL-R after 60 months. Therefore, it is assumed that in this cohort, a focal effect on the CR tissue due to abnormal loading in patients after ACL-R is not the primary cause of the worse clinical outcomes, but rather that the effect extends beyond the focal cartilage region and impacts the entire knee joint. The problem with clinical scores is that they do not represent the focal effect of the treated area after CR, but the global assessment of the knee joint. Therefore, MR morphological and biochemical assessment of the treated area is crucial when comparing CR procedures.

The development of osteoarthritis after ACL tear and even after ACL-R is often discussed: in a consensus project from 2022 Petersen *et al*.^6^ acknowledged the higher risk of OA after ACL tear with 100% agreement. They also recommended, that with ACL-R surgery, the risk of developing post-traumatic OA can be reduced.^6^ On the contrary, a 2014 systematic review suggested that there was no significant evidence that ACL-R is an effective intervention for the prevention of knee osteoarthritis.^3^ Although some randomized trials have compared ACL-R to conservative treatment with respect to long-term OA development—including studies with 10 to 15 years of follow-up^7,8^—these populations differ from ours in focus and design. However, our 5-year data already show emerging clinical differences between patient groups, which may reflect early manifestations of the joint health trajectories described in those longer-term studies.

As shown in other studies, the incidence of post-traumatic osteoarthritis after ACL-R is relatively high and while ACL-R has not been conclusively shown to slow the progression of osteoarthritis, several studies suggest that it may reduce the incidence of secondary meniscal and cartilage injuries.^6^ Although the finding of inferior PROMs in the ACL-R group is consistent with prior literature, our study adds a new perspective by evaluating focal CR outcomes using high-resolution morphological and compositional MRI—including T2 mapping—over a 5-year period. Notably, despite inferior clinical outcomes, the quality and integration of the repaired cartilage tissue remained comparable between groups, suggesting that the CR itself is structurally successful, even in the presence of prior ACL-R.

Duijvenbode *et al*.^21^ showed that patients (*n* = 19) after ACL reconstruction can benefit from CR with an improvement in IKDC and KOOS scores at 2 years, with significant differences in the IKDC Score and KOOS quality of life score. Comparable findings were reported by Amin *et al*.^22^ Our long-term data support these earlier results: all PROMs improved significantly in the ACL-R group over 60 months, although the gains were significantly smaller than in patients without ACL-R. This highlights a clinically relevant distinction: while patients with ACL-R can still achieve meaningful mid-term functional improvement following focal CR, their long-term outcomes diverge. Specifically, none of the PROMs in the ACL-R group exceeded the established PASS thresholds,^23^ whereas all scores in the no-ACL-R group did. This divergence underscores the importance of distinguishing structural repair success from overall patient-perceived knee function, particularly in the context of altered joint biomechanics following ACL-R. Focal CR may effectively restore tissue quality at the defect site—its primary therapeutic goal—but it is not designed to address global joint function. Our findings support that CR fulfills this focal objective, as the quality and integration of the repaired tissue remained unaffected by prior ACL-R. Although long-term knee function remained compromised in the ACL-R group, likely due to joint-wide alterations, the focal cartilage lesion itself did not show structural inferiority. This suggests that patients with prior ACL-R can still derive benefit from CR, particularly by preserving focal joint integrity and potentially mitigating further cartilage deterioration. This is especially relevant given prior evidence showing that untreated cartilage lesions at the time of ACL-R are associated with a progression to more severe cartilage damage over time.^5^ Our findings can guide clinicians in setting realistic expectations for patients undergoing CR post-ACL-R, particularly regarding the risk of premature OA and the long-term benefits of CR.

There are some limitations in this study: due to the retrospective nature of this study, it was not possible to evaluate the exact time point of ACL-R, but ACL repair was performed before, during, or within 6 weeks of cartilage treatment. Timing of these 2 procedures could be an important approach: Mehl *et al*. showed that simultaneous ACL-R and autologous chondrocyte implantation resulted in similar clinical outcomes compared to isolated ACI with intact ligaments.^19^ No focus was placed on a distinct time difference between ACL-R and CR and whether these altered biomechanical properties influenced CR. This underscores the need for future research to explore whether simultaneous or staged procedures yield better outcomes.

There were several other limitations: of the 112 patients originally enrolled, 71 were included in the final analysis. Patients with incomplete MRI data or missing clinical scores at follow-up were excluded to ensure consistency of data across outcome measures. While this reduced the overall sample size, it was necessary to maintain data quality and comparability. The potential for selection bias due to this exclusion represents a limitation of the study. A control group of ACL-R patients without CR was not available due to the retrospective design and original trial structure, limiting causal inference. BMI could not be analyzed due to the retrospective character. Retrospective clinical data on prior meniscal injuries or surgeries were not available. Although our inclusion criteria limited meniscal damage to a maximum of 50% resection, it is important to acknowledge that patients in the ACL-R group may have had more complex injury patterns, including a higher prevalence of partial meniscal injuries, which represents a potential confounding factor in interpreting between-group differences. However, follow-up MRIs demonstrated no meniscal tears in either group. The ACL-R group was significantly younger and more often male than the no-ACL-R group, which may reflect typical ACL injury demographics but introduces baseline heterogeneity. Also, no evaluation of the ACL-graft itself was performed. Other limitations of this study are that it was conducted at multiple centers and with different MRI systems; however, standardized MRI protocols were followed at all sites.

This study demonstrates that ACL-R does not negatively affect the structural quality or integration of repaired cartilage tissue following CR surgery, based on 5-year MRI-based morphological and compositional assessments. Although patients with ACL-R had significantly worse clinical scores at long-term follow-up, they still showed statistically significant improvements in all PROMs from baseline to 60 months. These results suggest that CR can offer mid-term functional improvement even in the presence of prior ACL reconstruction. However, due to the study’s retrospective nature and group heterogeneity, this should not be interpreted as a definitive indication for CR in all ACL-R cases. Instead, the findings highlight the importance of further prospective studies to determine whether—and under what conditions—patients with ACL-R benefit meaningfully from CR beyond focal tissue healing.
